# Case Report: Calpainopathy Presenting After Bone Marrow Transplantation, With Studies of Donor Genetic Content in Various Tissue Types

**DOI:** 10.3389/fneur.2020.604547

**Published:** 2021-01-11

**Authors:** Kristina Martens, Jamie Leckie, Daniel Fok, Robyn A. Wells, Sameer Chhibber, Gerald Pfeffer

**Affiliations:** ^1^Department of Clinical Neurosciences, Cumming School of Medicine, Hotchkiss Brain Institute, University of Calgary, Calgary, AB, Canada; ^2^Division of Neurology, Department of Medicine, Kelowna General Hospital, Kelowna, BC, Canada; ^3^Department of Medical Genetics, Alberta Child Health Research Institute, University of Calgary, Calgary, AB, Canada

**Keywords:** myopathy, calpain 3, LGMD2A, late onset, transdifferentiation, bone marrow transplantation, digital droplet pcr, dystrophin

## Abstract

We present a patient who had two allogeneic bone marrow transplantations for acute lymphocytic leukemia. She developed slowly progressive limb-girdle weakness in the context of other symptoms of graft-vs.-host disease (GVHD). Her myopathy symptoms had been initially attributed to GVHD, but when she progressed despite immunotherapy, genetic testing was requested. Initial testing was performed on a blood sample, identifying a variant of unknown significance in *DMD*. Subsequent testing of DNA from the patient's muscle tissue identified two pathogenic variants in *CAPN3*, with absence of the *DMD* variant (this latter variant presumed to have been received from the donor). Allele-specific digital droplet qPCR permitted the quantification of the donor variant in various tissues from the patient (whole skin, isolated fibroblasts, whole blood, saliva, buccal cells, urine sediment, and two muscle biopsies taken at a 2 year interval). This report emphasizes that genetic disease should still be considered in the context of presumably acquired disease, and also demonstrates the extent of transdifferentiation of donor cells into other tissues.

## Introduction

Genetic myopathies and muscular dystrophies can be difficult to diagnose on account of genetic and/or clinical heterogeneity, poor genotype-phenotype correlation, and variability in onset age. A compounding factor in late-onset muscle disease is the fact that patients may have coexistent acquired diseases that complicate the clinical picture and present additional diagnostic challenges. In general, medical diagnosis attempts to attribute symptoms to a unifying diagnosis, but in the modern era of genomic sequencing it is increasingly well-known that coincident rare disease diagnoses are common rather than being exceptional, either as being a case of dual genetic diagnoses (1), gene modifier effects (2), genetic disorders having very late onset (3), or combinations of genetic and acquired disease (4).

We present a case of a patient who was diagnosed with acute lymphocytic leukemia (ALL) and received two bone marrow transplants (BMT). She developed slowly progressive limb-girdle weakness which was initially attributed to graft-vs.-host disease (GVHD), however she did not respond to immunotherapy. We describe her genetic investigations (using DNA isolated from blood and muscle tissue) as well as genetic studies identifying donor DNA in various fractions in different tissues. The case emphasizes the need to consider genetic disease even in the context of other acquired diseases. This report also provides quantification of donor DNA within various tissue types.

## Methods

### Ethics Statement

Written informed consent was obtained from the patient for these studies and for publication of this case report. Ethical approval was obtained from the University of Calgary Conjoint Health Research Ethics Board.

### Clinical Chart Review

Retrospective chart review was conducted to report the evolution of the clinical history, therapies, and investigations, including two separate muscle biopsy procedures.

### DNA Extraction Protocols

Whole blood collected in EDTA tubes was isolated using a MagMAX DNA Multi-Sample Ultra 2.0 Kit (cat#A36570) on the KingFisher Duo Prime Purification System by ThermoFisher. Two skeletal muscle biopsies (collected in 2013 and 2016) were flash frozen in liquid nitrogen and stored at −80 until processed. A partial depth skin punch biopsy was collected and stored in Ham's F12 media (supplemented with 20% FBS, 1% pen/strep, 1% Glutamax, and 1% Fungizone) and kept at 4 degrees until processed. A portion of the skin biopsy was used for fibroblast isolation using a standard derivation of Primary Human Fibroblasts protocol. After collection, urine was centrifuged, washed with PBS and stored at −20 until processed. Muscle, skin, fibroblast and urine samples were isolated using the Qiagen All Prep DNA/RNA Mini Kit (Cat. 80204). Saliva and Buccal samples were collected with Oragene DNA kits, and gDNA isolated using prepIT L2P reagent by DNA Genotek.

### Exome Sequencing and Bioinformatics

Detailed methods are provided in Supplement. To summarize, library preparation used the Agilent SureSelect XT2 Target Enrichment System for Illumina Paired-End Multiplexed Sequencing. This protocol used reagents from the #G9621B SureSelect XT2 Reagent Kit and exome capture probes from the #5190-9501 SureSelect XT2 Clinical Research Exome V2 Capture Library. Sequencing was performed on an Illumina NextSeq 500 sequencer and 300 cycle (2 × 150 bp) high-output sequencing kits to produce ~10 Gb of sequence data (≥Q30) per sample. Variant calls were generated based upon the detailed protocol described in Supplement. The variant list was filtered to identify coding variants with MAF <0.005 from genes associated with neuromuscular diseases.

### PCR and Sanger Sequencing

Endpoint PCR and Sanger sequencing was used to screen for the patient's Calpain 3 germline mutations *CAPN3* c.550delA p.Thr184fs (rs80338800), and c.865C > T p.Arg289Trp (rs528417986), as well as *DMD* c.3028G > GC p.Ala1010Pro (rs766325631). We used custom primers for each variant (all primer sequences available on request). PCR was performed using Qiagen Taq polymerase, 400nM primer, 1ul of gDNA, 56°C annealing and 39 cycles. Amplified products were purified using ExoI/SAP, and Sanger Sequencing was performed using BigDye (Applied Biosystems) on an ABI 3730XL sequencer (Applied Biosystems). Chromatographs were interpreted using Mutation Surveyor software (Applied Biosystems).

### Allele-Specific Digital Droplet PCR (ddPCR)

To determine the proportion of genomes containing the *DMD*c.3028G > GC variant in different tissues, we performed allele-specific ddPCR using primers specific to the variant and reference sequences at the 3′ end. ddPCR reactions consisted of 12.5 ul EvaGreen Supermix (Bio-Rad cat#1864034), 400 nM primer, 50 ng gDNA, and nuclease free water up to 25 ul volume. Reactions were prepared in 96 well plates and transferred to an Automated Droplet Generator (Bio-rad) to create 20,000 droplets or individual PCR reactions. PCR Cycling conditions were carried on Bio-Rad C1000 Touch Thermal Cycler as follows: 95°C for 10 min, 45 cycles of 95°C for 30 s, 63°C for 1 min and a final extension of 98° for 10 min.

Droplet counts were analyzed using QX200 Droplet Reader (Bio-Rad) and QuantaSoft Analysis Pro. The relative proportion of genomes containing the variant was approximated by direct comparison of positive droplets in the variant specific reaction compared to the reference sequence specific reaction.

## Results

### Case Report

At time of referral the patient was a 38 year old woman with slowly progressive symmetric proximal weakness and muscle cramps. She had no prior history of neuromuscular disease. Four years prior, she was diagnosed with acute lymphocytic leukemia (ALL), and received two allogeneic BMTs (four and 2 years prior to presentation) from different donors. Following the second transplant she was in remission from ALL but developed eruptions affecting her skin and oral mucosa that were attributed to graft-vs.-host disease (GVHD). She originally received treatment with prednisone and tacrolimus for 1 month, and due to lack of effect tacrolimus was changed to cyclosporine. Prednisone was discontinued after another 9 months following which her GVHD symptoms remained well-controlled on cyclosporine.

Four months after stopping prednisone, she developed weakness when trying to rise from a chair, lifting arms over her head, and had symmetric muscle cramps. She was not reporting other neurological symptoms, namely no facial weakness, bulbar symptoms, contractures, cardiac or respiratory symptoms. Since she had not developed other GVHD symptoms, it was not clear whether these symptoms were a manifestation of GVHD or another condition. On family history, there were no family members known to have any neuromuscular condition. Her parents were non-consanguineous, and healthy in their 60′s. She had no siblings, and one son aged 12 who was healthy with normal development.

Her initial neurologic examination indicated normal cranial nerves, normal motor tone, bulk, and reflexes, and grade 4 MRC weakness in neck flexors, deltoid, biceps, triceps, hip flexors and abductors bilaterally, with normal strength in other muscle groups. Coordination and sensory examinations were normal. A myositis antibody panel including HMG-CoA-R and SRP antibodies were negative. EMG and nerve conduction studies were normal. Muscle biopsy of vastus lateralis was largely non-specific and unremarkable, with no inflammatory cell infiltrates, but possible rare regenerating myofibres.

On speculation that the patient may still have an immune-mediated myopathy, she received a trial of therapy with intravenous immunoglobulins and prednisone, followed by a trial of rituximab, which did not result in any improvement. Her cyclosporine was switched to tacrolimus which similarly did not provide any improvement.

**Figure 1:**
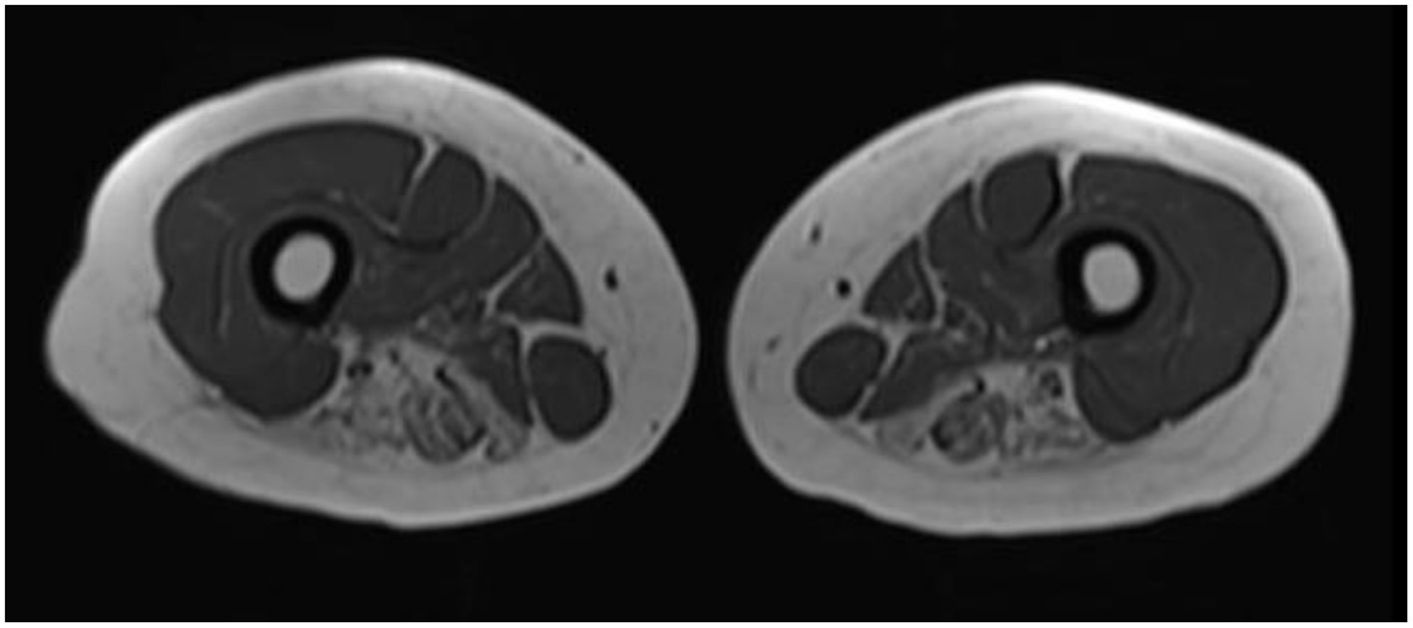
Muscle MRI of upper legs. In this representative transverse axial T1 weighted image from the patient, increased signal intensity is seen in the bilateral adductor magnus, semitendinosus, and semimembranosus muscles.

She was followed clinically and 2 years later her exam had deteriorated. She had developed scapular winging bilaterally and had atrophy developing in limb girdle musculature. Weakness had become MRC grade 4 in shoulder abduction, elbow flexion/extension, and knee flexion; MRC grade 2 strength was present in hip abductors/extensors. Given this change and a lack of clear diagnosis she was re-investigated, including a second muscle biopsy which was again inconclusive, with minimal myofibre size variation and occasional atrophic fibers. Dystrophin immunohistochemistry was normal. MRI of the upper legs showed fatty infiltration of posterior hamstring muscles with relative sparing of anterior leg compartment, iliopsoas, hip flexors (Figure 1).

A review of prior records identified an elevated CK of 890 U/L from prior to her onset of muscle symptoms, raising the question of a possible pre-existing genetic muscle disease. That CK elevation was recorded when she presented for investigation of chest pain, although ultimately her cardiac workup (troponin, ECG, and echocardiogram) had returned normal. A repeat CK level 2 days later had decreased to 510, but was still elevated (reference range <190U/L). At her initial presentation with myopathy symptoms, her CK level was 813 U/L, and subsequent measurements fluctuated from 834 to 1,522 U/L in her first year of these symptoms. In her second year of myopathy symptoms, the CK enzyme level ranged from 720 to 2,571 U/L. In both years she had received the above-mentioned immunosuppressive therapies, which did not result in clinical improvement or reliable reductions on CK levels. After discontinuation of these therapies her CK enzyme levels did not significantly change, and her most recent CK levels ranged from 672 to 1,835 U/L.

Testing with a genetic sequencing panel for 78 genes (5) associated with myopathy identified a heterozygous variant of unknown significance in *DMD* [c.3028G > GC, predicted to cause p.(Ala1010Pro), and listed in dbSNP as rs766325631], although testing had been performed on DNA isolated from blood, due to a test ordering error. Chimerism studies in the patient's blood revealed 100% chimerism for the second BMT donor, indicating that this variant actually originated from the second donor.

### Exome Sequencing of DNA Isolated From Muscle

Based on our variant prioritization approach we identified eight candidate variants. Compound heterozygous variants in *CAPN3* were identified [c.865C > T, predicted to cause p.(Arg289Trp), rs528417986 in dbSNP; and c.550delA, predicted to cause p.(Thr184fs), rs80338800 in dbSNP]. Both variants are listed as pathogenic in ClinVar. This was considered to be the explanation for the patient's condition given the characteristics of the variants, and the strong consistency of the clinical phenotype and muscle MRI with calpainopathy. However, we could not formally confirm that the variants are in trans because other family members were not available for testing.

The other variants identified from exome sequencing included six single heterozygous variants in *AGL, ATL1, MAP3K20, PDK3, TOR1AIP1*, and *TRAPPC11*. None of these were deemed as explanations for the patient's condition due to the inheritance pattern (recessive disease for *AGL, AMP3K20, TOR1AIP1*, and *TRAPPC11*) and/or phenotype associated with these genes (*ATL1* associated with dominant sensory neuropathy or spastic paraplegia; *PDK3* associated with X-linked dominant sensorimotor neuropathy).

As a matter of research interest we sought to identify any reads containing the *DMD* variant from testing from blood, but there were only three reads at this position and the variant was not present.

### Allele-Specific Digital Droplet PCR

**Figure 2:**
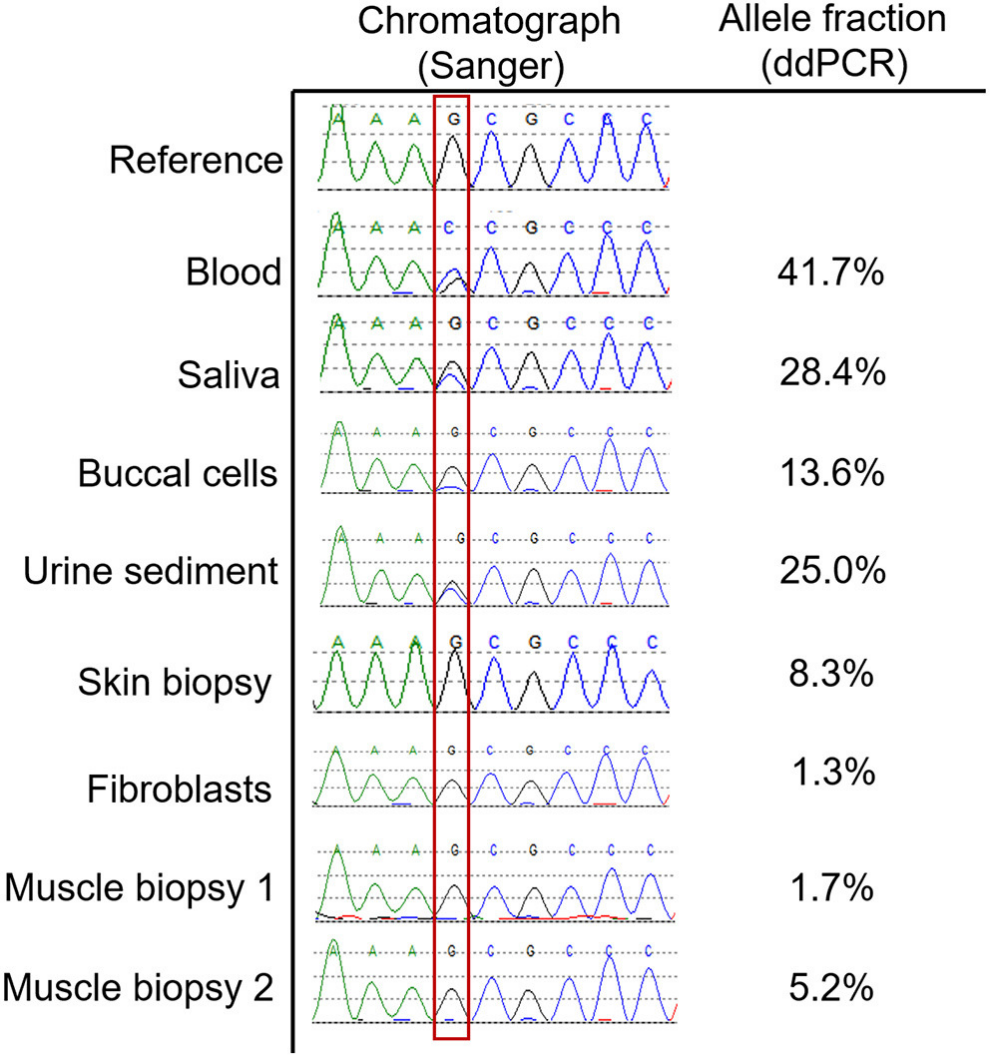
Allele fractions of the DMD variant in various tissue types. This figure shows the Sanger sequencing chromatographs at the position of the DMD variant (outlined with red box) in various tissue types, which is quantified using the described allele specific ddPCR assay. Blood predictably demonstrated an allele fraction that was approaching 50%. For tissue types that do not contain significant numbers of white blood cells, such as skin, fibroblasts, and muscle, the allele fractions were below 10%. For other tissues that contain a high proportion of white blood cells such as saliva, urine sediment and buccal cells, the allele fractions were intermediate.

In order to identify the extent to which the *DMD* variant may have transdifferentiated into other tissues, we performed allele-specific ddPCR on DNA from available tissue types including both muscle biopsies, a skin biopsy, urine sediment, buccal cells, and saliva. The results are presented in Figure 2, and demonstrate that the variant is present in a fairly large proportion of genomes isolated from buccal, blood, and urine sediment DNA sources. Skin and muscle tissue had detectable levels of the *DMD* variant but this was <10% in these tissues.

## Discussion

We describe a case of calpainopathy with late onset, that was a diagnostic challenge on account of the patient's diagnosis of ALL, two BMTs, and prior history of GVHD. In retrospect, the clinical phenotype is typical for calpainopathy, but this was difficult to recognize given that acquired disease was initially suspected in this context.

The c.550delA mutation is one of the common pathogenic mutations in *CAPN3* and has been associated with a range of phenotypes including LGMD as well as hyperCKaemia (6). The deleterious effect of this variant is presumably due to the frameshift and presumed loss of function. The c.865C > T mutation is located within the first insertion sequence (in the second protease core domain of CAPN3) (7). The insertion sequence contains two autolysis sites and we speculate that this mutation may affect autoproteolysis of CAPN3, which is a described mechanism for the pathogenicity of some CAPN3 mutations. For purposes of genotype-phenotype correlation, we are not aware of this combination of mutations in previously reported patients with calpainopathy.

This case provides several interesting learning points, the most important being that genetic disease should be considered in the differential diagnosis even when presenting with late onset disease and in the context of other medical comorbidities. In this particular case, it is possible that the medical comorbidities and medical therapy (particularly the long term steroid treatment) may have accelerated the course of this patient's genetic myopathy, which had previously been asymptomatic. There is no means to be certain whether an interaction could have occurred, though CAPN3-related disease covers a broad clinical spectrum ranging from asymptomatic hyper-CKaemia (8) to severe limb girdle weakness presenting in childhood (9), and in rare cases cardiomyopathy (10). The fact that wide phenotypic variability exists even within families with the same mutations (11) suggests that environmental or other circumstantial factors may play a role in the eventual outcome of the disease. Therefore, the steroid therapy, or the coexistent medical stressors (ALL and two BMTs) could have contributed to this patient's disease course, although her development of myopathy symptoms at the same time as her BMT treatments and GVHD could also have been coincidental.

Donor cells from BMT may transdifferentiate into other host tissues (12, 13), to varying extents up to 10% of the cell population (14). Given this, we speculated that if substantial transdifferentiation occurred, this would introduce the VUS in *DMD* from the donor, which could have a damaging effect on the recipient's muscle if this variant is pathogenic. Alternatively, transdifferentiation of donor cells could potentially have a beneficial effect, given the host's original cell population contains compound heterozygous mutations causing muscular dystrophy. In this case study, we demonstrate the extent of donor DNA in various tissue types, which is smaller in tissues such as muscle and skin, and higher in tissues which will have a contribution from white blood cells, such as saliva, buccal cells, and urine sediment. Our study demonstrates that these latter tissue sources may not be reliable for testing of genetic disease in individuals who have had BMT, which is useful information because DNA from saliva is increasingly used in clinical testing, and urine sediment is a highly useful source of DNA for particular genetic diagnoses (15).

We are aware of two prior examples in which a bone marrow transplant occurred in a patient with a genetic disease. In one case, the patient had dual genetic diagnoses of dystrophinopathy as well as X-linked severe combined immunodeficiency (16). BMT was used to treat the immune deficiency, and subsequent studies of muscle biopsies up to 13 years later revealed persistence of transdifferentiated donor cells, in the range of 0.5–0.9%. The second report was a dual genetic diagnosis of dystrophinopathy and Diamond-Blackfan anemia (17). Subsequent muscle biopsies on days 730 and 1,250 post-BMT revealed 8 and 10.4% chimerism, respectively, in the recipient's muscle tissue. Results from our study are similar to that present in the second report, if one considers that the allele fraction of the DMD variant constitutes only 50% of the donor DNA (given that the variant was heterozygous in the donor). We have summarized these findings in Table 1.

**Table 1:** Comparison of bone marrow donor genetic material in muscle tissues compared with prior literature.

**Publication**	**Donor genetic content (%)**	**Time from BMT to muscle biopsy (years)**
Current report	3.4	2
	10.4	5
Gussoni et al. (16)	0.5–0.9	13
Nair et al. (17)	8	2
	10.4	3

*This table summarizes the extent of donor genetic content within muscle biopsy tissues in prior reports compared to this study. Each of the reports applied different methods, although the findings in the current study are similar to that reported in one of the prior studies. Note that the donor genetic content was multiplied by two compared to the result from Figure 2, because our ddPCR assay was specific for a variant that was heterozygous in the donor*.

In conclusion, this study emphasizes the importance of considering genetic disease in the differential diagnosis of late-onset presentations even in the presence of medical comorbidities, suggests possible environmental factors which could be explored in future studies of calpainopathy, emphasizes the preferred tissue sources for genetic testing, and contributes to the highly limited literature about the transdifferentiation of BMT cells into tissues such as muscle.

## Data Availability Statement

The datasets generated in this article are not openly available because of ethical concerns which could potentially allow the participant to be identified. Requests to access the datasets should be directed to the corresponding author.

## Ethics Statement

The studies involving human participants were reviewed and approved by University of Calgary Conjoint Health Research Ethics Board. The patients/participants provided their written informed consent to participate in this study. Written informed consent was obtained from the patient for the publication of any potentially identifiable images or data included in this article.

## Author Contributions

KM analysis of laboratory data and authored manuscript. JL, DF, and SC collection of clinical data and drafted sections on clinical data. RW analysis of laboratory data. GP conceptualization of study, supervision of laboratory studies, and authored manuscript. All authors approved the final version of the manuscript for submission.

## Conflict of Interest

The authors declare that the research was conducted in the absence of any commercial or financial relationships that could be construed as a potential conflict of interest.
