# Supplementary material for: Case Report: Calpainopathy Presenting After Bone Marrow Transplantation, With Studies of Donor Genetic Content in Various Tissue Types
Source: Front Neurol. 2021 Jan 11;11:604547. doi: 10.3389/fneur.2020.604547 (PMC7829329; doi:10.3389/fneur.2020.604547)
Supplement: Supplementary file 1 [file Data_Sheet_1.docx]

Detailed methods:

Exome sequencing:

Genomic DNA samples (100 ng) were sheared to ~ 150 bp fragments using a Covaris S2 sonicator. The fragments were then converted into dual-indexed Illumina compatible sequencing libraries according to the Agilent SureSelect XT2 Target Enrichment System for Illumina Paired-End Multiplexed Sequencing Protocol. This protocol used reagents from the #G9621B SureSelect XT2 Reagent Kit and exome capture probes from the #5190-9501 SureSelect XT2 Clinical Research Exome V2 Capture Library. In this protocol the DNA samples were separately end-repaired, 3’-adenylated, and indexed by PCR amplification with Agilent pre-capture indexing adaptors. Exome capture was then performed on pools of eight samples (187.5 ng each) using the exome capture library and 24 hour hybridization (65^o^). Hybridized DNA was captured on streptavidin beads, eluted and then PCR amplified (8 cycles) a final time. The final pools of eight exomes were verified by size analysis (Agilent TapeStation D1000 assay) and qPCR (Kapa qPCR Library Quantitation Kit).

Illumina sequencing was performed by combining the exome samples in three pools (total of 24 exomes) into a single 2.1 pM solution. An Illumina NextSeq 500 sequencer and 300 cycle (2x150 bp) high-output sequencing kits were then used, as per the Illumina sequencing protocol, to produce approximately 10 Gb of sequence data (>= Q30) for each exome sample.

Bioinformatics:

Sample demultiplexing and conversion to FASTQ files was performed using Illumina’s bcl2fastq software v2.18.0.12. FASTQ formatted reads were aligned to the GRCh37 human reference genome plus the mitochondrial revised Cambridge reference sequence using BWA (v. 0.8.15) in minimum exact match mode with default parameters, then sorted and stores as BAM files using samtools v1.6. Sequencing reads from putative PCR duplicate fragments were marked using Picard (v2.8.3) MarkDuplicates, followed by BuildBamIndex. Subsequent steps performed using GATK are based on v3.7-0-gcfedb67 and default parameters except where noted. GATK was used to identify regions that were candidates for realignment using RealignerTargetCreator, followed by IndelRealigner. Base qualities were recalibrated using GATK’s BaseRecalibrator with dbSNP v138 as a SNP truth set, and Mills and the 1000 Genomes as insertion and deletion truth sets. Recalibration sanity was reviewed by manual inspection of the GATK AnalyzeCovariates output plots.  Final BAMs were generated using GATK PrintReads using the base recalibration output.

The deduplicated and realigned BAMs were initially genotyped as follows. Each sample had initial variants called (gVCF files generated) using the GATK HaplotypeCaller. Pooled haplotyping was then performed using GATK GenotypeGVCFs functionality (with non-standard useNewAFCalculator option), including all exomes from the sequencing run, and a database of HaplotypeCaller gVCFs from 160 unrelated exomes (including the NA12878 genome-in-a-bottle trio) that had previously been run on the same NextSeq 500 using the same exome capture kit. The GATK VariantAnnotator function was used to generate statistics for downstream variant quality calibration, specifically: QualByDepth, StrandOddsRatio, FisherStrand, BaseQualityRankSumTest, MappingQualityRankSumTest, ReadPosRankSumTest and InbreedingCoeff.  SNPs were recalibrated with GATK’s VariantRecalibrator and ApplyCalibration using the following truth training sites: HapMap v3.3 and Genome-In-A-Bottle trio (per Illumina Platinum Genomes) with phred-scaled prior probability of 15, Omni v2.5 with prior 12, 1000 Genomes phase 1 high confidence SNP calls with prior 10. The dbSNP v138 sites were not used for training, but were used as known sites with a prior of 2 for GATK’s VCF filter field tranche stratification (using thresholds 100, 99.9, 99 and 90). Insertions and deletions were also recalibrated with GATK’s VariantRecalibrator and ApplyCalibration using the following truth training sites: Genome-In-A-Bottle trio, and Mills and 1000 Genomes gold standard indels with prior 12, and known sites for stratification based on dbSNP v138 insertions and deletions with prior 2.

The pooled genotyped VCF file was analyzed for related samples using PLINK2 to generate a BED file of variants with a minimum genotype quality phred-scaled value of 30, and minimum read depth of 10 per sample. KING was used to generate a sample kinship matrix, which was converted (using a custom script) to a PLINK PED file for downstream use in GATK. Final genotype VCFs for each sample or group of related samples as appropriate were generated using GATK’s SelectVariants. VCFs containing multiple related samples were then processed using GATK PhaseByTransmission, followed by GATK VariantAnnotator for PossibleDeNovo and VariantFiltration to annotate low quality de novo calls with genotype quality <20.

The final VCF genotype files were annotated for coding variant prediction using SnpEff v4.3k with database GRC37.75, for functional predictions using SnpSift v4.3k with database dbNSFP2.9, and for population frequencies using Annovar  v2017-07-17 with data from GnomAD v1.0.2 (both genome and exome) and Genomes Middle East.
